# A GLM-based zero-inflated generalized Poisson factor model for analyzing microbiome data

**DOI:** 10.3389/fmicb.2024.1394204

**Published:** 2024-05-30

**Authors:** Jinling Chi, Jimin Ye, Ying Zhou

**Affiliations:** ^1^School of Mathematics and Statistics, Xidian University, Xi'an, China; ^2^School of Mathematical Sciences, Heilongjiang University, Harbin, China

**Keywords:** factor analysis, GLM, microbiome data, zero inflation, ZIGP model

## Abstract

**Motivation:**

High-throughput sequencing technology facilitates the quantitative analysis of microbial communities, improving the capacity to investigate the associations between the human microbiome and diseases. Our primary motivating application is to explore the association between gut microbes and obesity. The complex characteristics of microbiome data, including high dimensionality, zero inflation, and over-dispersion, pose new statistical challenges for downstream analysis.

**Results:**

We propose a GLM-based zero-inflated generalized Poisson factor analysis (GZIGPFA) model to analyze microbiome data with complex characteristics. The GZIGPFA model is based on a zero-inflated generalized Poisson (ZIGP) distribution for modeling microbiome count data. A link function between the generalized Poisson rate and the probability of excess zeros is established within the generalized linear model (GLM) framework. The latent parameters of the GZIGPFA model constitute a low-rank matrix comprising a low-dimensional score matrix and a loading matrix. An alternating maximum likelihood algorithm is employed to estimate the unknown parameters, and cross-validation is utilized to determine the rank of the model in this study. The proposed GZIGPFA model demonstrates superior performance and advantages through comprehensive simulation studies and real data applications.

## 1 Introduction

The human microbiome is the collection of all microorganisms that live in and associate with the human body, including bacteria, archaeobacteria, protists, and viruses, distributed in the nasal cavity, oral cavity, skin, gastrointestinal tract, and genitourinary tract. The growing significance of the microbiome in ecosystems is increasingly recognized. In particular, the relationship between gut microorganisms and human health has garnered widespread scientific interest. Over time, an increasing number of studies have demonstrated that dysbiosis of the gut microbial community is associated with complex diseases, such as human gastrointestinal disorders (Willing et al., 2010; Machiels et al., 2013; Knights et al., 2014), metabolic traits, diabetes (Turnbaugh et al., 2006; Wen et al., 2008; Vijay-Kumar et al., 2010), obesity (McKnite et al., 2012; Carlisle et al., 2013; Parks et al., 2013), and inflammatory bowel disease (Frank et al., 2007). These investigations significantly contribute on exploring the causes and treatments of diseases. Furthermore, complex interactions between hosts and microbiota are also observed in various ecosystems. For example, in marine ecosystems, microbial communities associated with seaweed play an vital role in the development, reproduction, function, and defense of seaweeds (Egan et al., 2013; Singh and Reddy, 2016). Therefore, it becomes crucial to quantify the abundance of microbial taxa and investigate the association between microbiota and diseases or traits.

The development of high-throughput sequencing (HTS) technology has been widely employed in microbial research, enabling researchers to identify the composition and abundance of microbial species directly (Kuczynski et al., 2011). Microbiome data are typically generated by extracting samples from the specified environment, followed by sequencing the 16S rRNA genes of the DNA extracts using high-throughput sequencing technology. The obtained sequence reads are compared with the reference 16S rRNA database and assigned to Operational Taxonomic Units (OTUs) based on a sequence similarity threshold (e.g., 97%; Tyler et al., 2014). High-throughput sequencing data provide valuable insights for investigating the relationship between the microbiome and the host environment or clinical factors. As a motivating application, we consider the gut microbiome data in Sun et al. (2019), which explores the association between gut microbes and obesity. The authors sequenced 16S rDNA genes of 48 individuals and obtained a dataset with 895 OTUs, where the number of variables (i.e., OTUs) vastly exceeds the number of observations (i.e., the number of samples). Moreover, we found that ~45% of the OTU counts were zero, and the variance of the data significantly exceeded the mean. These characteristics are manifestations of high dimensionality, zero inflation, and over-dispersion, which may distort downstream analysis. However, many microbiome datasets exhibit the same problems as the motivating data, posing challenges for statistical analysis. Firstly, most microbiome data are non-negative counts with a large number of zeros (i.e., zero-inflated; Xu et al., 2015; Kaul et al., 2017). Some of these observed zeros result from insufficient sequencing depth (i.e., library size, which is the total number of reads obtained by per sample from equipment) or other technical reasons that result in some taxa not being detected, and others are the fact that some taxa are very rare and not present in most samples (Silverman et al., 2020). Traditional statistical methods may not accurately estimate the parameters of the data distribution due to the preponderance of zeros, leading to biased results (Campbell, 2021). Secondly, microbial abundance data only represent relative information in observed samples and cannot describe the abundance in the entire ecosystem (Mandal et al., 2015; Gloor et al., 2017). Moreover, the sequencing depth varies among samples, and even the variation between samples is magnitude (Sims et al., 2014). Finally, microbiome data are typically over-dispersed and high-dimensional (Kurtz et al., 2015; Xu et al., 2015; Armstrong et al., 2022). The number of taxa in the OTUs table may significantly exceed the number of observed samples, which is a sign of high dimensionality. The high dimensionality of the data may strain computational resources and increase the risk of overfitting. Meanwhile, the standard model may underestimate the true variation within the data when over-dispersion exists, leading to inaccurate estimation and hypothesis testing (Robinson et al., 2009; Love et al., 2014). Detecting associations between microbes and diseases remains challenging because of the complex features of microbiome data and the limitations of current statistical methods. Therefore, it is necessary to develop novel statistical analysis methods for the characteristics of microbiome data.

Zero-inflation and over-dispersion of count data have received widespread attention from scholars recently. Wagh and Kamalja (2017) briefly reviews different zero-inflated models for handling count data and the performance of their parameter estimation, which provides suggestions for selecting parameter estimation methods for zero-inflated models. Motivated by zero-inflation and over-dispersion problems, a zero-inflated negative binomial (ZINB) mixed regression approach is proposed to analyze the data on the length of stay for pancreas disorder (Yau et al., 2003). However, in a few cases, the parameter estimation algorithm for the ZINB regression model fails to converge (Lambert, 1992). A zero-inflated generalized Poisson (ZIGP) regression model has been proposed to model domestic violence data with too many zeros (Famoye and Singh, 2006). It is a strong competitor to the Poisson and negative binomial regression model when the count data is over-dispersed. In addition, zero-inflated generalized Poisson and zero-inflated negative binomial regression models were used in QTL mapping studies for the count traits with excess zeros (Cui and Yang, 2009; Moghimbeigi, 2015; Chi et al., 2020). More recently, Tirozzi et al. (2022) used zero-inflation models to assess long-term population trends and elucidate the effects of environmental bias, over-dispersion, and zero-inflation on the population trend estimates. These studies provide some inspiration for analyzing microbiome data with complex characteristics.

In recent years, extensive research has been conducted by scholars to address the challenges associated with microbiome data, including zero inflation, high dimensionality, and over-dispersion (Zhang et al., 2018; Xu et al., 2020; Jiang et al., 2023). Two typical methods have been proposed to address the zero-inflated structure of sequencing data. One method is replacing the zeros with small non-zero positive number (pseudo count; Chen and Li, 2013; Lin et al., 2014). However, the effects of creating pseudo count has not been evaluated thoroughly when the data contain excessive zeros. Besides, the choice of pseudo count may impact subsequent analysis (Costea et al., 2014), and this approach is not statistically rigorous. Moreover, the idea of multiplicative replacement has been proposed. The non-parametric replacement method can be used to adjust the data through multiplicative modification under simple conditions (a small number of zeros; Martín-Fernández et al., 2003). In other cases, more sophisticated model-based methods can be utilized to replace zeros in the data (Martín-Fernández et al., 2012). Recently, a Bayesian-multiplicative treatment has been proposed to solve the problem of count zero, which assumes a Dirichlet prior for the proportions and replaces the zeros with posterior Bayesian estimates (Martín-Fernández et al., 2014). The other standard and widely used method is to construct a two-part model with a point probability mass at zero along with another parametric distribution, such as zero-inflated Gaussian model (Xu et al., 2015), zero-inflated lognormal model (Sohn et al., 2015), zero-inflated Poisson model (Xu et al., 2020), zero-inflated negative binomial model (Jiang et al., 2019; Zhang and Yi, 2020), and many others (Peng et al., 2016; Tang and Chen, 2018; Zeng et al., 2022; Jiang et al., 2023). The advantage of this method is that an appropriate model can be selected according to the nature of the data. For example, the zero-inflated negative binomial model can effectively address the issue of zero-inflated and over-dispersion in the data because the negative binomial provides a standard statistical model for over-dispersed data.

The other well-known challenge for analyzing microbial data is the high dimensionality of the data. Generally, the number of taxa usually far exceeds the observed samples in the OTUs table, which is a symbol of high dimensionality (Armstrong et al., 2022). Therefore, dimensionality reduction technology is used to map high-dimensional data into a potential low-dimensional space while retaining the primary information in the data intact to facilitate the subsequent analysis, which is a desirable preprocessing step (Fan et al., 2015; Jasner et al., 2021).

Factor analysis, an extensively employed technique, serves as a prominent method for dimensionality reduction of high-dimensional data. Pierson and Yau (2015) proposed a zero-inflated factor analysis (ZIFA) model to explicitly consider excess zeros in Single-cell RNA-seq data. However, the ZIFA model preprocesses count data via a normal transformation, which may overlook its inherent count nature and potentially result in information loss during the preprocessing step. Lee et al. (2013) developed a Poisson factor model with offsets to explicitly incorporate the special features that count nature and heterogeneous library size (the total reads per sample). Subsequently, the negative binomial factor regression model was proposed to reduce the dimensionality of microbial abundance data, and then model the associations of microbial abundance and host-associated features by including only a subset of the predictors for a few latent factors (Mishra and Müller, 2022). However, these two methods (Poisson factor model and negative binomial factor model) are only suitable for data that does not contain excessive zeros, which fail to consider zero inflation. Sohn and Li (2017) proposed a GLM-based ordination method for microbiome samples (GOMMS), which employs a zero-inflated quasi-Poisson factor model to dimensionality reduction and overcome the challenge of zero-inflation. However, this method assumes that each taxa has a fixed probability of zero, which is generally not easily satisfied. More recently, Xu et al. (2020) proposed a factor analysis model based on the zero-inflated Poisson distribution (ZIPFA), which can more flexibly adapt to some characteristics of microbial data, such as count value, excessive zeros, and high dimensionality. A significant critique of Poisson models is the failure to accommodate over-dispersion, which has been widely observed for microbiome data. Following this line of research, we combined the zero-inflated generalized Poisson distribution with factor analysis under the framework of the generalized linear model to propose a GLM-based zero-inflated generalized Poisson factor analysis (GZIGPFA) model, which provides a valuable dimensionality reduction tool for microbiome data. The GZIGPFA model can also address the issues of over-dispersion and handle the zero-inflated structure. Furthermore, our method models absolute abundance directly, avoiding the information loss attributable to data transformation.

The rest of this paper is organized below. Section 2 presents a new GZIGPFA model for handling microbiome data and introduces methods for parameter estimation and rank selection. A simulation and comparison study are conducted in Section 3 to demonstrate the performance of the proposed method. In Section 4, we apply our method to the gut microbial data to explore the association between gut microbes and obesity. In Section 5, a conclusion of this paper is drawn with a discussion of extensions and areas for subsequent work.

## 2 Method

For *i* = 1, 2, …, *n* and *j* = 1, 2, …, *m*, let *y*_*ij*_ denote the count of the *j*-th taxon from the *i*-th individual, then, an *n*×*m* microbial abundance matrix can be expressed as ***Y*** = (_*y*_*ij*_)*n*×*m*_. Denote the *i*th row of matrix ***Y*** as ***y***_(*i*)_ = (*y*_*i*1_, …, *y*_*im*_), refer to as the *i*-th sample of sequencing data.

### 2.1 Zero-inflated generalized Poisson factor model

The microbiome dataset typically presents as a highly skewed non-negative count matrix with numerous zeros, often characterized by over-dispersion. Therefore, we build statistical models to address these issues for microbiome data.

The presence of zeros in microbiome data may be true absences or undetected taxa. Considering the over-dispersion characteristics of the data, we assume that the sequencing count *y*_*ij*_ follows the zero-inflated generalized Poisson (ZIGP) distribution (Famoye and Singh, 2006):


(1) $$y_{ij}\sim \{\begin{array}{ll} 0, & \text{with probability }\phi_{ij},\\ GP(T_{i}\lambda_{ij},\alpha ), & \text{with probability }1-\phi_{ij}, \end{array}$$

where *ϕ*_*ij*_ is the zero-inflation parameter describing the probability of excess zero; *GP*(*T*_*i*_λ_*ij*_, α) is the generalized Poisson distribution (Consul and Famoye, 1992; Famoye, 1993), with the probability function


(2) $$\begin{array}{l} p\left(y_{ij};T_{i}\lambda_{ij},\alpha \right)=\frac{1}{y_{ij}!}{\left(\frac{T_{i}\lambda_{ij}}{1+\alpha T_{i}\lambda_{ij}}\right)}^{y_{ij}}{\left(1+\alpha y_{ij}\right)}^{y_{ij}-1}\text{exp}\\ \left\{-\frac{T_{i}\lambda_{ij}\left(1+\alpha y_{ij}\right)}{1+\alpha T_{i}\lambda_{ij}}\right\}, \end{array}$$

where λ_*ij*_ and α are the mean and dispersion parameters of the generalized Poisson part, respectively; *T*_*i*_ is the relative library size of the *i*-th sample, which is utilized to regulate λ_*ij*_. Generally, there are many representations of *T*_*i*_ (Anders and Huber, 2010; Eddy, 2011; Badri et al., 2020; Mishra and Müller, 2022). In this paper, we take


$$T_{i}=\sum_{j=1}^{m}y_{ij}/\text{median}\left(\sum_{j=1}^{m}y_{1j},\ldots ,\sum_{j=1}^{m}y_{nj}\right).$$

Next, the link between the zero-inflation probability *ϕ*_*ij*_ and the mean parameter λ_*ij*_ is established according to Lambert (1992). Typically, an increase in the number of zeros in the data results in a smaller overall mean. Therefore, a negative relationship between *ϕ*_*ij*_ and λ_*ij*_ is established, i.e.,


(3) $$\begin{array}{l} \text{logit}\left(\phi_{ij}\right)=-\tau \log\left(\lambda_{ij}\right), \end{array}$$

where τ is the shape parameter; logit(*ϕ*_*ij*_) and log(λ_*ij*_) are the link functions for the probability of zero-inflation and the mean of generalized Poisson in the generalized linear model (GLM), respectively. Let $\Lambda ={\left(\lambda_{ij}\right)}_{n\times m}\in {\mathbb{R}}^{n\times m}$ and $\Phi ={\left(\phi_{ij}\right)}_{n\times m}\in {\mathbb{R}}^{n\times m}$ be the matrix forms of λ_*ij*_ and *ϕ*_*ij*_, respectively. Therefore, the matrix form of Equation (3) can be expressed as logit(**Φ**) = −τlog(**Λ**).

The ZIGP model (Equation 1) described above can accommodate simultaneously zero inflation and over-dispersion count data. Furthermore, upon review of the existing literature about microbiome data analysis, the ZIGP model represents the inaugural utilization of the zero-inflated generalized Poisson model in microbiome datasets. In the following, we intend to solve the prevalent issue of high dimensionality in the microbiome data with a factor analysis model. Therefore, we propose a GLM-based zero-inflated generalized Poisson factor analysis (GZIGPFA) model to provide a suitable model for zero-inflated, over-dispersed, and high-dimensional microbiome data.

Assume that matrix log(**Λ**) has a low-rank structure log(**Λ**) = ***FL***^*T*^ with rank *K* (Lee et al., 2013), where ***F***∈ℝ^*n*×*K*^ is the factor score matrix and $F={\left(f_{\left(1\right)}^{T},\ldots ,f_{\left(n\right)}^{T}\right)}^{T}$ with ***f*****_(*i*)_** = (*f*_*i*1_, …, *f*_*iK*_), *i* = 1, 2, …, *n*; ***L***∈ℝ^*m*×*K*^ is the loading matrix and $L={\left(l_{\left(1\right)}^{T},\ldots ,l_{\left(m\right)}^{T}\right)}^{T}$ with ***l***_(*j*)_ = (*l*_*j*1_, *l*_*j*2_, …, *l*_*jK*_), *j* = 1, 2, …, *m*. Then, we consider the following zero-inflated generalized Poisson factor model:


(4) $$\{\begin{array}{l} y_{ij}\sim \text{ZIGP}(T_{i}\lambda_{ij},\alpha ,\phi_{ij}),\\ \text{logit}(\phi_{ij})=-\tau \log(\lambda_{ij}),\\ \log(\lambda_{ij})=f_{i1}l_{j1}+f_{i2}l_{j2}+\ldots +f_{iK}l_{jK}, \end{array}$$

where *f*_*ik*_ is an element of the matrix ***F***, denoting the *k*th factor score for the *i*-th sample; *l*_*jk*_ is an element of matrix ***L***, denoting the loading of the *j*th taxon on the *k*th factor, where *i* = 1, 2, …, *n*, *j* = 1, 2, …, *m*, and *k* = 1, 2, …, *K*. In this model, the logarithm is the canonical link function in the generalized linear model (GLM) framework (McCullagh and Nelder, 1989).

After the rank *K* is determined and the unknown parameters α, τ, ***F***, ***L*** in the model (Equation 4) are estimated, we reduce the dimensionality of the microbiome dataset from *m* to *K*. The score matrix ***F*** possesses an equivalent sample size to the original microbiome dataset ***Y*** but only has *K* variables. In subsequent work, it is easier to perform association analysis between disease phenotypes and the low-dimensional score matrix, providing a brief tool for investigating the relationship between microbiome and disease.

### 2.2 An alternating maximum likelihood algorithm

To estimate the unknown parameters α, τ, ***F***, ***L*** in model (Equation 4), we adopt a method that maximizes the ZIGP likelihood function:


(5) $$\begin{array}{l} \begin{array}{c} L\left(\alpha ,\tau ,F,L\right)=\overset{n}{\underset{i=1}{\prod }}\overset{m}{\underset{j=1}{\prod }}\left[\phi_{ij}I_{\left\{y_{ij}=0\right\}}+\left(1-\phi_{ij}\right)p\left(y_{ij};T_{i}\lambda_{ij},\alpha \right)\right], \end{array} \end{array}$$

where *p*(*y*_*ij*_; *T*_*i*_λ_*ij*_, α) is the probability function of GP distribution (Equation 2); $\log\left(\lambda_{ij}\right)=\overset{K}{\underset{k=1}{\sum }}f_{ik}l_{jk}$ and logit(*ϕ*_*ij*_) = −τlog(λ_*ij*_). In Equation (5), α, τ, ***F***, ***L*** are the unknown parameters, and it is challenging to maximize the likelihood directly. Therefore, we consider an alternating maximum likelihood algorithm in the GLM framework to estimate the parameters.

In order to obtain the initial ***F*** and ***L***, we apply the singular value decomposition (SVD) to the log-transformed matrix $\tilde{Y}$ and obtain the singular, i.e., $\log\left(\tilde{Y}\right)=U\Sigma V^{T}$. Set ***L***^*old*^ = *V*^*T*^ and $F^{old}=\left(U_{\left(,1\right)}\Sigma_{11},U_{\left(,2\right)}\Sigma_{22},\ldots ,U_{\left(,K\right)}\Sigma_{KK}\right)$, where Σ_*kk*_, *k* = 1, …, *K* is the *k*th diagonal element of Σ.

***Step 1***: Assuming that factor score matrix ***F*** is known as ***F***^*old*^, a ZIGP regression model is fitted with the *j*th column of matrix ***Y*** (denoted by ***y***_*j*_) as the response and ***F***^*old*^ as a covariate matrix, the regression model can be written as


$$\{\begin{array}{l} y_{j}\sim \{\begin{array}{ll} 0, & \text{with probability }\phi_{j},\\ GP(T\lambda_{j},\alpha ), & \text{with probability }1-\phi_{j}, \end{array}\\ \log(\lambda_{j})=F^{old}l_{\left(j\right)}^{T},\\ \text{logit}(\phi_{j})=-\tau \log(\lambda_{j}), \end{array}$$

where the vector ***T*** = (*T*_1_, *T*_2_, …, *T*_*n*_) is the relative library size vector; the regression coefficient vector ***l***_(*j*)_ = (*l*_*j*1_, *l*_*j*2_, …, *l*_*jK*_) is the *j*th row of the factor loading matrix $L^{new}={\left(l_{\left(1\right)}^{T},l_{\left(2\right)}^{T},\ldots ,l_{\left(m\right)}^{T}\right)}^{T}$; the vectors **λ**_*j*_ and **ϕ**_*j*_ are the *j*th column of the matrices **Λ** and **Φ**, respectively.

To estimate the unknown parameter vector $\theta ={\left(\tau ,\alpha ,l_{\left(j\right)}\right)}^{T}$ of the regression model, we should maximize the likelihood function. However, the explicit solution of each parameter cannot be obtained by directly using the maximum likelihood estimation method. Therefore, we perform parameter estimation of the regression model with the EM algorithm. The detailed procedure of the EM algorithm is given in Appendix A.

Since matrix ***Y*** has *m* columns, we need to fit *m* GLMs to obtain *m* rows of ***L***^*new*^. However, the proposed model assumes that the τ and α remain the same across all *m* different GLMs. To accommodate this, we combine ***y***_1_, …, ***y***_*m*_ into a column vector and solve all *m* models simultaneously to obtain the globally optimal τ and α values.

After estimating τ, α and ***L***, we continue to update ***F***. The process is similar to Step 1.

***Step 2***: Fit a ZIGP regression model with the *i*th row of matrix ***Y*** (denoted by ***y***_(*i*)_) as the response and the estimated loading matrix ***L*** = ***L***^*new*^ from the previous step as a covariate, the regression model can be written as


$$\{\begin{array}{l} y_{\left(i\right)}\sim \{\begin{array}{ll} 0, & \text{with probability }\phi_{\left(i\right)},\\ GP(T_{i}\lambda_{\left(i\right)},\alpha ), & \text{with probability }1-\phi_{\left(i\right)}, \end{array}\\ \log(\lambda_{\left(i\right)})=f_{\left(i\right)}L^{newT},\\ \text{logit}(\phi_{\left(i\right)})=-\tau \log(\lambda_{\left(i\right)}), \end{array}$$

where *T*_*i*_ is the relative library size of the *i*th sample; the regression coefficient vector ***f***_(*i*)_ = (*f*_*i*1_, *f*_*i*2_, …, *f*_*iK*_) is the *i*th row of the factor score matrix $F^{new}={\left(f_{\left(1\right)}^{T},f_{\left(2\right)}^{T},\ldots ,f_{\left(n\right)}^{T}\right)}^{T}$; the vectors **λ**_(*i*)_ and **ϕ**_(*i*)_ are the *i*th row of the matrices **Λ** and **Φ**, respectively. Next, the parameters α, τ, and ***f***_(*i*)_ in the regression model are estimated by the EM algorithm. The specific process is similar to Step 1.

Since matrix ***Y*** has *n* rows, we need to fit *n* GLMs to obtain *n* rows of ***F***^*new*^. However, the proposed model assumes that the τ and α remain the same across all *n* different GLMs. Therefore, similar to step 1, we combine ***y***_(1)_, …, ***y***_(*n*)_ into a column vector and solve all *n* models simultaneously to obtain the globally optimal τ and α values.

***Step 3***: Apply the singular value decomposition (SVD) method to the ***F***^*new*^***L***^*newT*^ to obtain a new ***F***^*old*^, and repeat the above alternating algorithm until convergence.

When the percentage of total likelihood difference between two iterations is less than a certain small value, the algorithm terminates; otherwise, we continue to update ***F***, ***L***, τ and α until convergence. In the ZIGP regression step, we will use the EM algorithm to estimate the parameters. Therefore, the likelihood increases due to the nature of the EM algorithm used in regression estimation (Dempster et al., 1977; Wu, 1983). The likelihood remains the same in the SVD step. Overall, the algorithm is guaranteed to converge. In the Step 3, we apply SVD to ***F***^*new*^***L***^*newT*^, which ensures the uniqueness and the orthogonality of the updated components. We briefly summarize the alternating maximum likelihood algorithm under the GLM framework in the “Algorithm 1” box.

**Algorithm 1:**
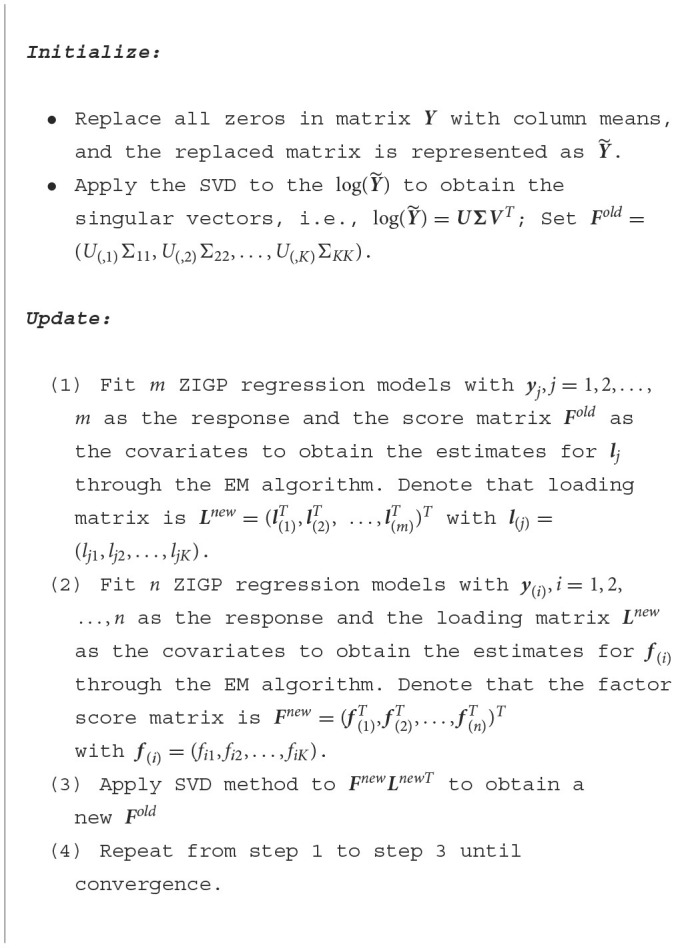
GZIGPFA algorithm.

### 2.3 Rank estimation

We use the *N*-fold cross-validation suggested by Li et al. (2018) to determine the optimal number of factors, i.e., the rank *K* of model (Equation 4). The idea is to randomly divide the entries of a data matrix into *N* non-overlapping parts. We systematically exclude one block at a time and utilize the remaining data to estimate the unknown parameters with varying ranks. Subsequently, we compute the likelihood of the model using the data of the excluded block. Finally, we sum up the likelihood of all *N* folds to obtain the total cross-validation (CV) likelihood of the model with rank *k* and calculate the CV likelihood for every rank *k*. The rank that provides the maximum CV likelihood is chosen as the optimal rank. The procedure of rank selection is briefly summarized in the “Algorithm 2” box.

**Algorithm 2:**
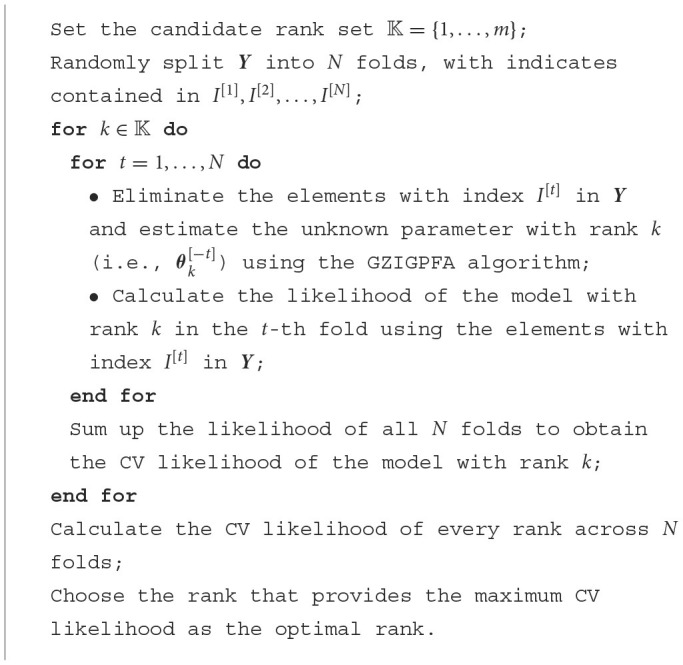
*N*-fold cross-validation for rank estimation.

## 3 Simulation studies

The performance of our proposed GZIGPFA method is demonstrated through a simulation study. We compare the GZIGPFA method with four other methods:

- ZIPFA (Zero-inflated Poisson factor analysis): This method uses a zero-inflated Poisson factor analysis model for reducing the dimension of the microbiome data while accommodating the zero-inflated nature of the data (Xu et al., 2020).
- log-PCA (log-principal component analysis): The data is preprocessed by replacing all zeros with a small value and then taking a logarithm of the transformed data. After that, the data is processed by performing a principal component analysis (PCA).
- PSVDOS (Poisson Singular Value Decomposition with Offset): The method is an efficient algorithm for estimating the Poisson factor model, which addresses the issue of sample normalization through the use of unknown offset parameters (Lee et al., 2013).
- GOMMS (GLM-based ordination method for microbiome samples): This method uses a zero-inflated quasi-Poisson factor model, which accounts for characteristics of microbiome data (e.g., highly skewed non-negative counts with excessive zeros) while reducing dimensionality (Sohn and Li, 2017).

### 3.1 Simulation design

**Figure 1:**
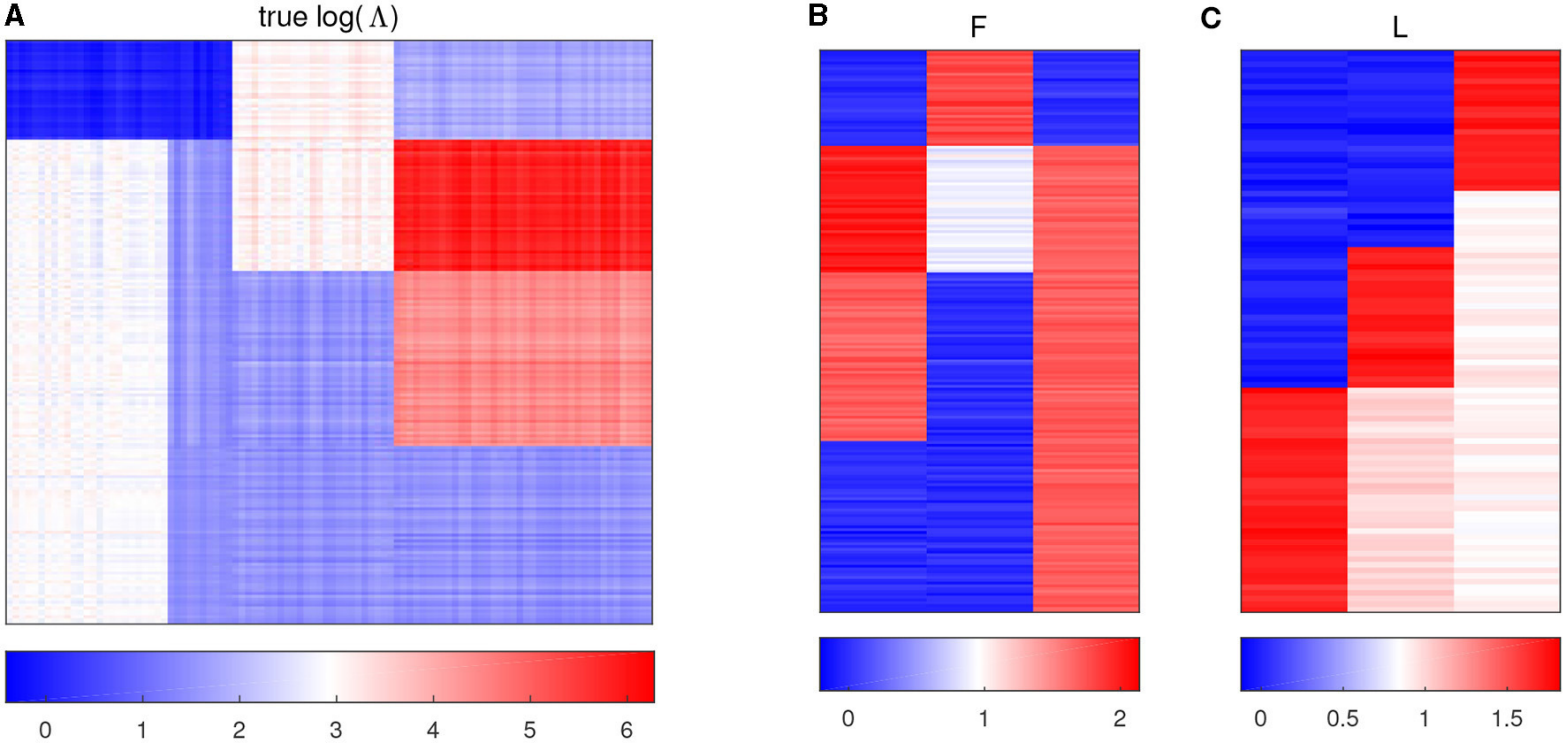
Plots of simulation parameters. **(A)** The heatmap of true log(**Λ**) matrix. The rows represent samples, and the columns represent taxa. **(B)** True left singular vector matrix ***F***. The rows correspond to the samples, and the columns denote factors. **(C)** True right singular vector matrix ***L***. The rows correspond to the taxa, and the columns denote factors.

We followed the design of Xu et al. (2020) to simulate microbiome datasets. A sequence data of *n* samples and *m* taxa is generated according to model (Equation 4). We simulate *n* = 200 different samples measured on *m* = 100 taxa. The rate matrix **Λ** follows: log(**Λ**) = ***FL***^*T*^, where the ***F***∈ℝ^*n*×3^ is a left singular vector matrix, and ***L***∈ℝ^*m*×3^ is a right singular vector matrix. To generate matrix ***F***, we create a 200-by-3 matrix ***F*** such that:

Column 1: *F*(36:80, 1) = 2, *F*(81:140, 1) = 1.7,

Column 2: *F*(1:35, 2) = 1.8, *F*(36:80, 2) = 0.9,

Column 3: *F*(1:35, 3) = 1.7, *F*(36:200, 2) = 0,

with all the other entries being 0, and then jitter all the entries by adding random noises generated from *N*(0, 0.06^2^). Similarly, to generate matrix ***L***, we create a 100-by-3 matrix ***L*** such that:

Column 1: *L*(1:60, 1) = 0, *L*(61:100, 1) = 1.7,

Column 2: *L*(36:60, 2) = 1.7, *L*(61:100, 2) = 1,

Column 3: *L*(1:25, 3) = 1.7, *L*(26:100, 2) = 0.9,

with all the other entries being 0, and then jitter all the entries by adding random noises generated from *N*(0, 0.05^2^). Figure 1A displays the heatmap of the true log(**Λ**) matrix, and the three columns of ***F*** and ***L*** are shown in the columns of Figures 1B, C, respectively. Each row in Figure 1B corresponds to one sample, and Figure 1C shows the heatmap of the right singular vector matrix ***L***, in which each row indicates one taxon profile.

After the matrices ***F*** and ***L*** are generated, **Λ** can be obtained according to **Λ** = exp(***FL***^*T*^). Next, a zero-inflated sequencing matrix ***Y*** was generated from the following ZIGP model,


$$f\left(y_{ij};\alpha ,\lambda_{ij},\phi_{ij},T_{i}\right)=\phi_{ij}I_{\left\{y_{ij}=0\right\}}+\left(1-\phi_{ij}\right)GP\left(y_{ij};T_{i}\lambda_{ij},\alpha \right),$$

where λ_*ij*_ is an element of the matrix **Λ**; the scaling parameter *T*_*i*_ and the dispersion parameter α were set to 1 and 0.2, respectively; the probability of excess zero *ϕ*_*ij*_ is obtained by establishing the link between *ϕ*_*ij*_ and λ_*ij*_. Firstly, we consider the scenario with the relationship between *ϕ*_*ij*_ and λ_*ij*_ established in Section 2, that is,

- *Scenario* 1: logit(*ϕ*_*ij*_) = −τ log(λ_*ij*_).

Furthermore, to more comprehensively evaluate the robustness of the proposed method, we further considered generating sequencing data under several misspecified scenarios. First, consider two common links to *ϕ*_*ij*_ and λ_*ij*_, which are mentioned in Lambert (1992) besides Scenario 1:

- *Scenario* 2: log{− log(*ϕ*_*ij*_)} = τ log(λ_*ij*_).
- *Scenario* 3: log{− log(1 − *ϕ*_*ij*_)} = τ log(λ_*ij*_).

In addition, we set up a scenario along the lines in Sohn and Li (2017), namely, each taxon has a fixed probability *ϕ*_*j*_ independent of the λ_*ij*_:

- *Scenario* 4: *ϕ*_*j*_ ~ Uniform(τ − 0.10, τ + 0.10).

Finally, considering that actual microbiome data may come from different distributions, we set up two misspecified scenarios for generating microbiome data from other distributions, that is, the data come from the ZIP and ZINB distributions, and the relationship between the *ϕ*_*ij*_ and λ_*ij*_ follows the setup in Scenario 1:

- *Scenario* 5: *y*_*ij*_ ~ ZIP distribution, and logit(*ϕ*_*ij*_) = −τ log(λ_*ij*_).
- *Scenario* 6: *y*_*ij*_ ~ ZINB distribution, and logit(*ϕ*_*ij*_) = −τ log(λ_*ij*_).

We evaluated the simulation results for all the scenarios above at light zero inflation (20%) and higher zero inflation (40%), respectively.

### 3.2 Simulation results

**Figure 2:**
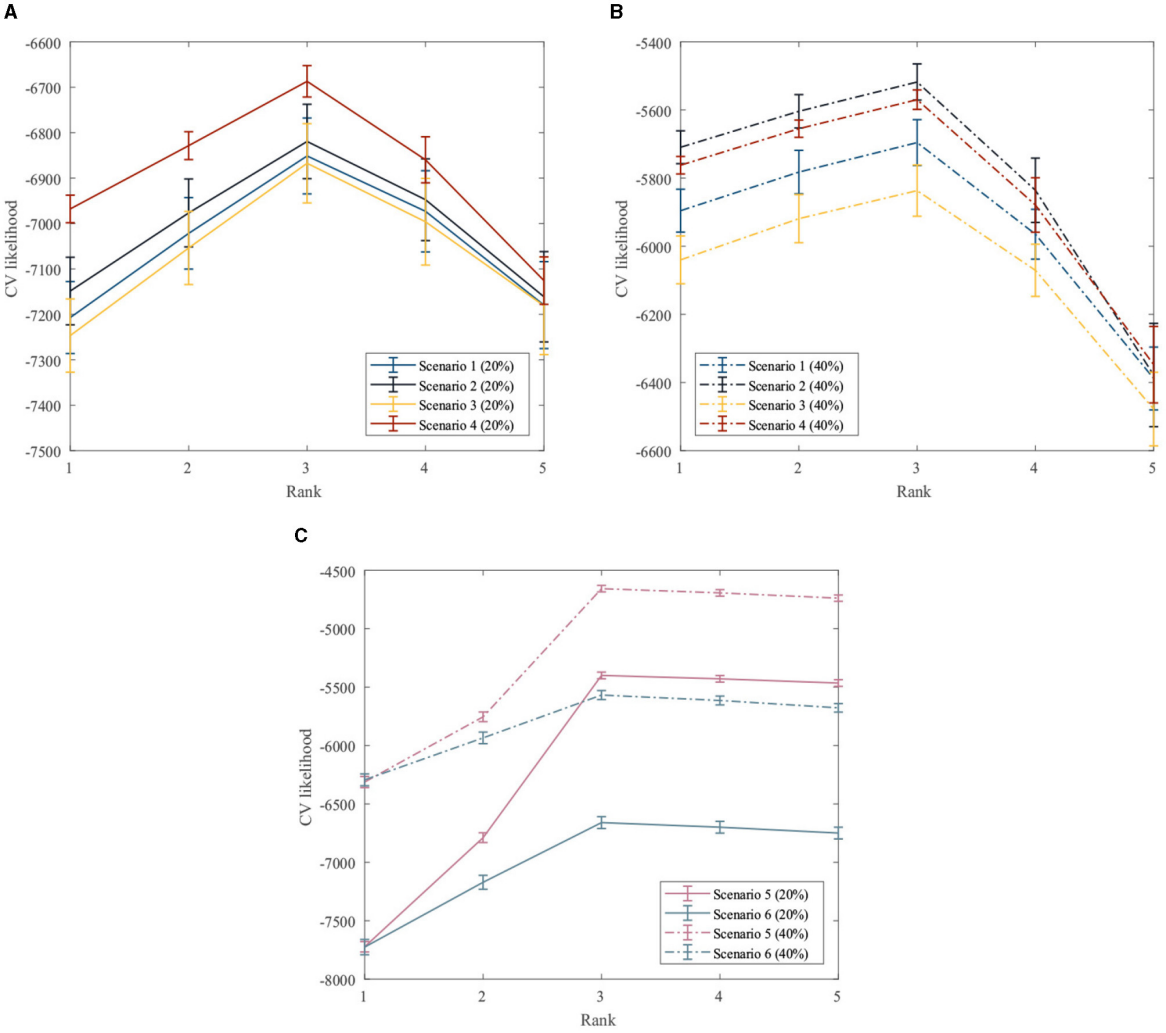
Cross-validation to choose the rank in the GZIGPFA model. **(A)** The CV likelihood of the GZIGPFA model under scenarios 14 with a 20% zero-inflated probability. **(B)** The CV likelihood of the GZIGPFA model under scenarios 14 with a 40% zero-inflated proportion. **(C)** The CV likelihood of the GZIGPFA model under scenarios 5 and 6 with 20% and 40% zero-inflated proportions. The proposed method provides maximum CV likelihoods with rank 3 under all simulation scenarios.

First, the performance of the proposed method for rank estimation in all scenarios is examined. A 10-fold cross-validation is performed on the data generated in each scenario separately to compute the cross-validation likelihood for different ranks. The rank estimation results of the GZIGPFA method for all simulation scenarios are displayed in Figure 2. It can be seen from Figure 2 that the proposed method provides the maximum CV likelihood with rank 3 in all simulation scenarios. Figure 2 shows that the proposed method is accurate in the rank estimation under the given model [Model (Equation 4)] and performs well under the misspecified scenarios, indicating the robustness of the GZIGPFA method.

Next, a comprehensive comparison of the GZIGPFA method with other approaches (ZIPFA, log-PCA, PSVDOS, and GOMMS) is presented to illustrate the superior performance of the proposed method in depth. For each simulation scenario, 200 replicates are performed. The Frobenius norm of the error matrix (denoted as loss value) is utilized to evaluate the effectiveness of each method. The loss values of several methods in all simulation scenarios are listed in Table 1. Table 1 shows that the GZIGPFA method has a small loss in most simulation scenarios, indicating that the proposed method is effective. In Scenarios (1)–(4), the performance of all four methods is significantly better than the GOMMS method. In addition, we find that the convergence effect of the GOMMS method is poor when there are more zeros in the data. In scenario (5), ZIPFA outperforms GZIGPFA when the zero percentage is high (40%) because this scenario essentially favors ZIPFA by using the ZIP model. In Scenario (6), the data is generated by the ZINB model, and the PSVDOS method performs the worst among the five methods because this method cannot consider over-dispersed and zero-inflated data. In addition, the GOMMS method performs second only to GZIGPFA and ZIPFA in Scenario (6) because GOMMS is based on the zero-inflated quasi-Poisson, which is intrinsically closer to ZINB. Overall, our method performs better than competing methods, even in the misspecified scenarios.

**Table 1:** The mean of loss values and standard errors (in the parenthesis) for the five methods under different scenarios.

**Scenario**	**Zero (%)**	**GZIGPFA**	**ZIPFA**	**log-PCA**	**PSVDOS**	**GOMMS**
Scenario (1)	20%	**6.9878** (1.4968)	10.7800 (1.8965)	10.2970 (0.1332)	26.4999 (0.0594)	92.5108 (207.740)
	40%	**12.7542** (7.1569)	13.2471 (2.9255)	16.7528 (0.1516)	26.6175 (0.0843)	102.689 (238.263)
Scenario (2)	20%	**8.9129** (3.2038)	10.8945 (2.0912)	10.7148 (0.1366)	26.5064 (0.0581)	87.5358 (166.145)
	40%	**10.2343** (4.8788)	13.5586 (3.3809)	17.7686 (0.1339)	26.7447 (0.1101)	91.5380 (144.401)
Scenario (3)	20%	**7.1095** (1.2251)	10.8162 (1.8302)	10.0293 (0.1294)	26.5015 (0.0644)	99.1790 (183.318)
	40%	21.5684 (8.9917)	**12.1517** (2.5438)	15.8540 (0.1635)	26.5817 (0.0819)	114.108 (243.236)
Scenario (4)	20%	**10.2995** (0.9843)	11.5437 (2.1585)	12.0547 (0.2186)	26.5496 (0.0694)	78.4083 (114.947)
	40%	**9.8594** (4.7380)	13.0524 (3.0187)	17.5302 (0.2297)	26.7168 (0.0957)	100.821 (241.874)
Scenario (5)	20%	**1.9069** (0.0882)	2.0074 (0.0686)	5.7243 (0.1271)	26.2521 (0.0052)	4.7897 (0.0036)
	40%	3.9383 (0.2266)	**3.2860** (0.2841)	13.0891 (0.1697)	27.2165 (0.2778)	10.5189 (0.0140)
Scenario (6)	20%	**2.9830** (0.1168)	3.3560 (0.2495)	6.4711 (0.1243)	26.2505 (0.0030)	6.0003 (0.0013)
	40%	**4.4550** (0.3200)	4.5013 (0.5164)	13.7113 (0.1683)	26.6065 (0.3625)	10.0941 (0.0107)

The best results in each setting are in boldface.

**Figure 3:**
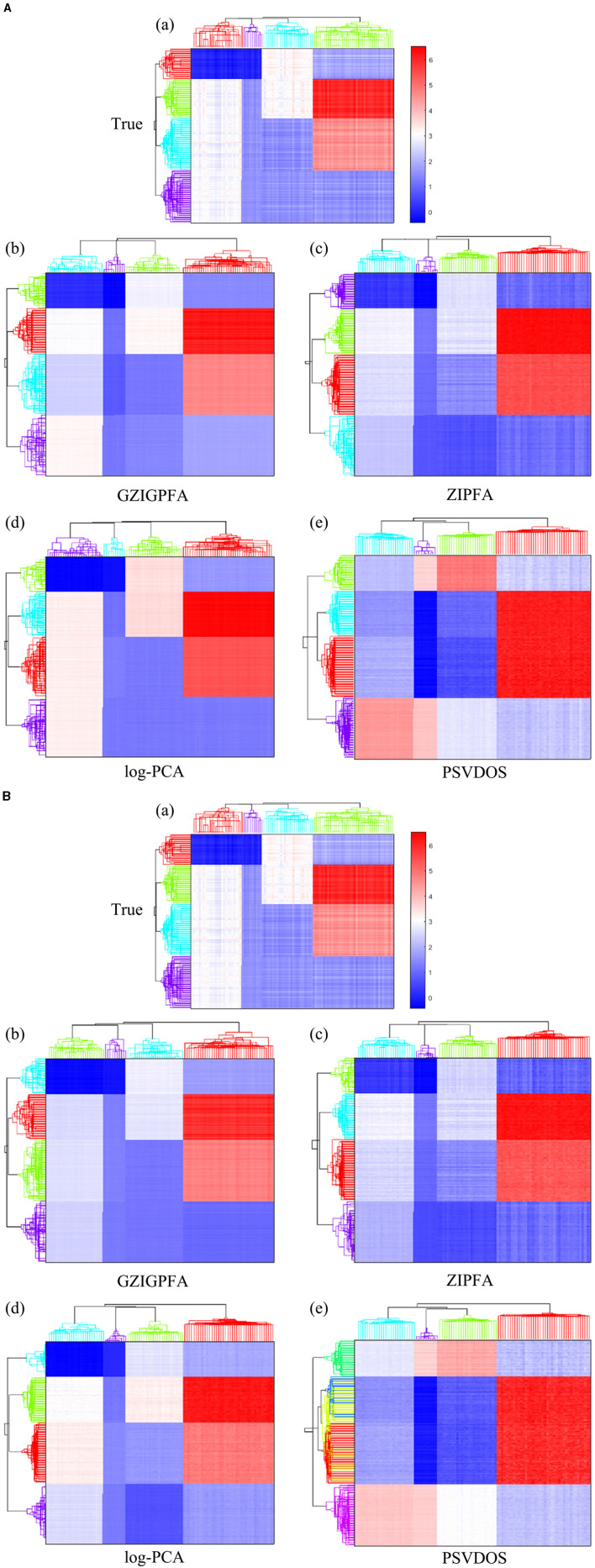
The heatmap of true log(**Λ**) and the estimated log(**Λ**) from different methods in Scenario 1. The phylogenetic tree on the top and left show the clustering of taxa and samples, respectively. **(A)** The zero-inflated proportion is 20%. **(B)** The zero-inflated proportion is 40%. The rows and columns of all heatmaps represent samples and taxa, respectively.

Finally, we show the heatmaps of the true log(**Λ**) and the estimated log(**Λ**) of several methods in Figure 3 to visualize the performance of the GZIGPFA method, and we also display the clustering effects of several methods at the taxa (top) and sample (left side) levels. Since the GOMMS method performs poorly in Table 1, only the estimation and clustering results of the four methods GZIGPFA, ZIPFA, log-PCA, and PSVDOS are presented in Figure 3. Panel (a) in Figures 3A, B displays the true log(**Λ**) used in the simulation. The phylogenetic tree on the left side of the heatmap shows the clustering of the sample, which falls into four clusters. Similarly, the phylogenetic tree above the heatmap shows the clustering of the taxa. The clustering pattern is obtained by applying the complete linkage hierarchical clustering analysis to ***F*** and ***L*** (Wilkinson and Friendly, 2009). Figures 3A, B show the estimation and clustering effects of the four methods when the zero-inflated proportion is low (20%) and high (40%) in Scenario (1), respectively. GZIGPFA method (Panel b in Figures 3A, B) offers the best approximation to the true signal, and it gives the accurate clustering result, which is as expected, as the dataset was designed in a way that takes advantage of the unique features of GZIGPFA. The log(**Λ**) estimated by the log-PCA method (Panel d in Figures 3A, B) is far from the true value (Panel a) and is the worst performer among several methods. Meanwhile, log-PCA fails to capture the right sample clustering when the zero-inflated proportion goes from 20 to 40%. The log-PCA performs poorly overall because it does not consider the underlying distribution and excessive zeros.

## 4 Application to the gut microbiome data

Empirical research has demonstrated that mice and humans harbor similar microbiota at high taxonomic levels (Ley et al., 2008; Krych et al., 2013). Therefore, laboratory mice can be used to simulate the human gut environment for experiments and to explore the mechanisms of host-microbial interactions in a data-driven manner when studying human gut microbes. In this section, the GZIGPFA model is applied to the mouse gut microbial dataset (Sun et al., 2019) to explore the association between gut microbes and obesity. Microbial datasets were extracted from 48 male mice. The mice were divided into the blank group, high-fat control group, and probiotic experimental group, in which the blank group was fed normal chow, the high-fat control group and the probiotic experimental group were fed a high-fat diet for 4 weeks to establish an obesity model for the mice, and the probiotic experimental group was fed high-fat chow plus probiotic capsules starting from the 5th week of the successful modeling, while the high-fat control group continued to be fed high-fat chow. At the end of the 8th week, various indicators of the mice were measured, including weight, body length, total cholesterol, endotoxin, etc. The samples were first amplified with a set of primers targeting the 16S rDNA V4 region. Then, the original data were subjected to operational taxonomic unit (OTU) clustering and species classification analysis based on valid data. According to the results of OTU clustering, species annotation was performed for the representative sequences of each OTU, and the corresponding species information and species-based abundance were obtained. Then, we reduced the dimensionality of the dataset with the proposed GZIGPFA method to extract the common factors and further explore the association between the common factors and obesity.

We selected body weight, total cholesterol, and endotoxin as three responses from the measured indicators of mice, where the weight of mice can intuitively reflect the degree of obesity. Obesity caused by a high-fat diet is often accompanied by hyperlipidemia, and total cholesterol (TC) is widely employed clinically as an indicator for measuring blood lipids. Endotoxin, also known as lipopolysaccharide (LPS), is a critical factor in the systemic inflammatory reaction. When the intestinal microbiota is imbalanced and harmful bacteria increase, the body is susceptible to endotoxemia, and sustained low-level endotoxemia is the leading cause of obesity and metabolic disorders. Therefore, we will focus on the relationship between the gut microbial community and three responses (weight, TC, and LPS).

**Figure 4:**
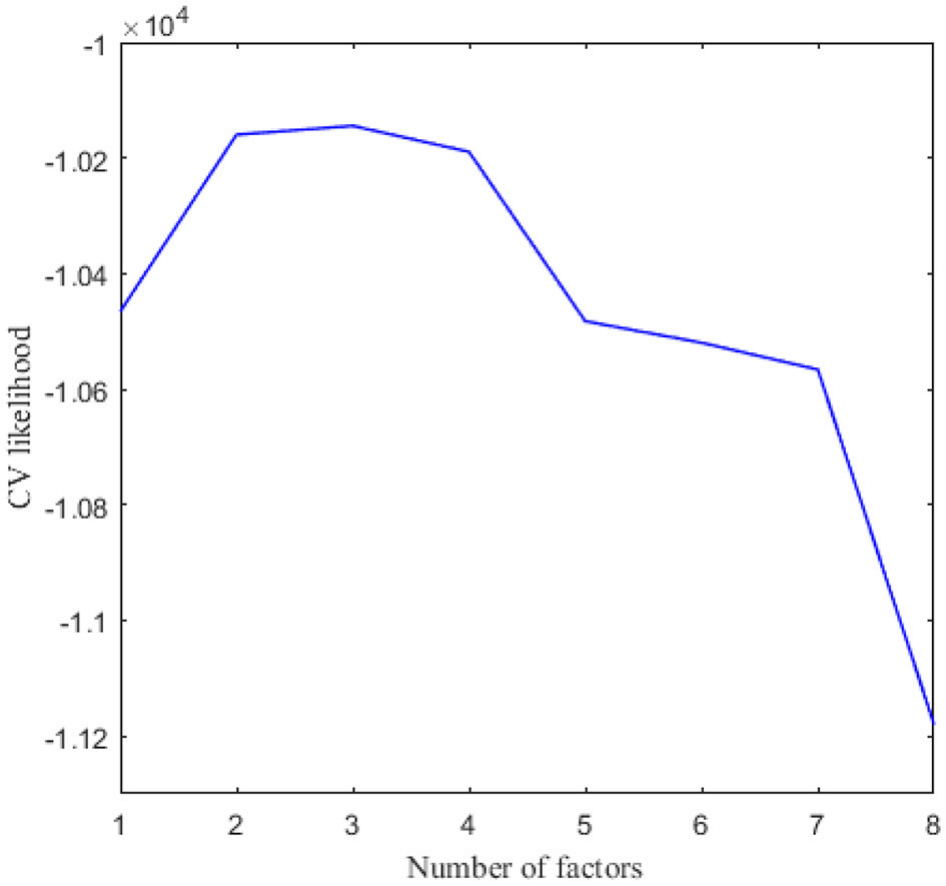
Cross validation to choose the number of factors in real data analysis.

**Figure 5:**
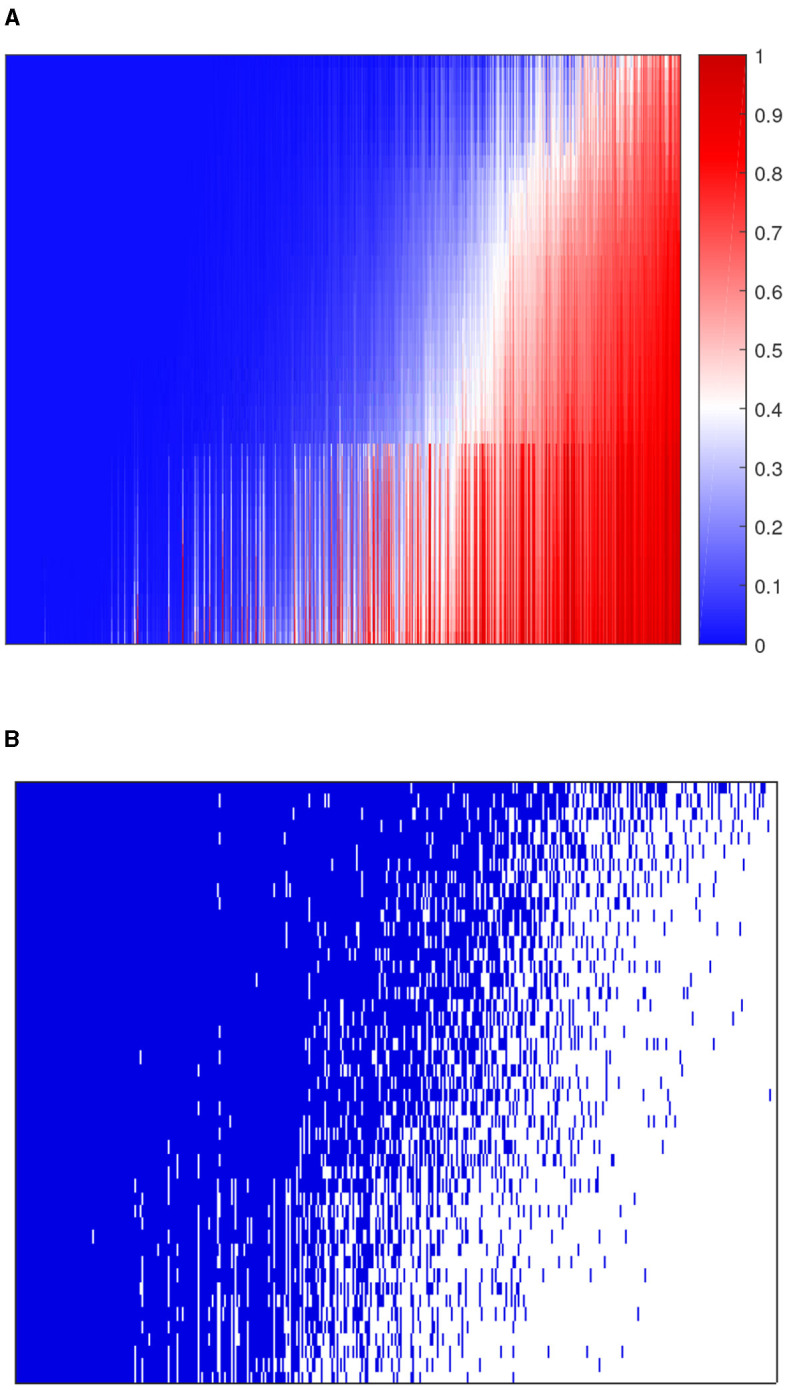
Comparison of predicted probability of zeros and real zero distribution in the dataset. **(A)** The heatmap of predicted zero probability. **(B)** The heatmap of the binary real data value. Blue points are non-zero values and white points are zeros. The rows and columns of both heatmaps represent samples and taxa, respectively.

We applied 10-fold cross-validation on microbial abundance data. Figure 4 shows that CV likelihood reaches the maximum point at a rank equal to 3, so we will use three factors in the following analysis. We performed GZIGPFA fitting with a rank of 3 on the microbiome data. The algorithm converged after 6 iterations and obtained score matrix estimates (***F***) and loading matrix estimates (***L***). We can compute log(**Λ**) = ***FL***^*T*^ according to the estimates of ***F*** and ***L***, and the zero-inflated probability matrix **Φ** can be obtained through the relationship between **Λ** and **Φ** assumed in Section 2.1 [i.e., logit(**Φ**) = −τlog(**Λ**)]. The total probability of zero for each count is estimated as ${\hat{\phi }}_{ij}+e^{-T_{i}{\hat{\lambda }}_{ij}/\left(1+\hat{\alpha }T_{i}{\hat{\lambda }}_{ij}\right)}$. We reorder the total zero probability matrix and plot the corresponding heatmap (Figure 5A). In Figure 5A, the bottom right indicates the large values of total zero probability (red points), and the small total zero probability values are sorted to the top left (blue points). Meanwhile, the rearrangement of the true data is plotted in Figure 5B, where non-zero values are shown in the top left (blue points) and zeros in the bottom right (white points). We compare the predicted probability of zeros with the distribution of zeros in the real data. The significant similarity between the red part in Figure 5A and the white part in Figure 5B shows that the proposed method captures the structure of excess zeros well.

To determine the association between the three factors obtained through GZIGPFA dimensionality reduction and the three responses (weight, TC, and LPS), a linear model was fitted in which each response was regressed on all three factors, respectively. The *p*-values corresponding to different factors and responses are listed in Table 2. In addition, we demonstrate the results of the other comparison methods (ZIPFA, log-PCA, PSVDOS, and GOMMS) introduced in Section 3. It can be observed in Table 2 that the GZIGPFA and log-PCA methods can identify factors significantly associated with each response, while ZIPFA and PSVDOS failed to provide significant factors for TC and LPS. In addition, GOMMS is also unable to find factors associated with LPS. In particular, all five methods identified factors (factors 2 or 3) associated with weight, indicating that gut microbiome composition may be an essential factor influencing weight. Furthermore, factor 2 was a significant predictor of all responses in our proposed method, suggesting a potential link between obesity diseases and gut microbiome.

**Table 2:** The *P*-values corresponding to different factors and response variables in different models.

**Response**	**Factor**	**GZIGPFA**	**ZIPFA**	**log-PCA**	**PSVDOS**	**GOMMS**
	Factor 1	0.2874	0.3727	0.3551	0.5600	0.0810
Weight	Factor 2	0.0004^***^	0.4077	0.0003^***^	0.0482^*^	0.9480
	Factor 3	0.0281^*^	0.0008^***^	0.0493^*^	0.1706	0.0260^*^
	Factor 1	0.1243	0.2850	0.5406	0.8740	0.0684
LPS	Factor 2	0.0171^*^	0.2530	0.0092^**^	0.1460	0.4635
	Factor 3	0.7297	0.1050	0.7876	0.660	0.2885
	Factor 1	0.4014	0.7030	0.8232	0.3290	0.0099^**^
TC	Factor 2	0.0076^**^	0.5972	0.0035^**^	0.1430	0.9905
	Factor 3	0.7657	0.0504	0.5464	0.6070	0.1704
	Factor 1	0.6075	0.0705	0.6760	0.7870	0.7837
TNF-α	Factor 2	0.7743	0.0873	0.6760	0.4050	0.5351
	Factor 3	0.9345	0.4159	0.5480	0.3520	0.2076

^***^, ^**^, and ^*^ denote the significance level takes 0.001, 0.01, 0.05, respectively.

We consulted the literature to explain the factors obtained by GZIGPFA. By searching for gut microbes and obesity keywords on PubMed, some review articles were screened to identify microbes related to obesity. To identify microbes associated with obesity by searching for gut microbes and obesity keywords on Pubmed and filtering some review articles. After a full-text review of 116 papers, Pinart et al. (2021) concluded that *Firmicutes* and *Bacteroidetes* are the two microorganisms that mainly affect obesity at the phylum level. Therefore, factors 2 and 3 significantly associated with the obesity phenotype in the association analysis may be summarized as *Firmicutes* and *Bacteroidetes*. In conclusion, the proposed method can help the experimenter to determine the approximate factors affecting the experiment in advance.

Finally, in order to demonstrate that the proposed model does the absence of over-prediction problems, we additionally selected a response for analysis, i.e., tumor necrosis factor-alpha (TNF-α), which is not directly related to obesity. As can be seen from Table 2, all these methods did not identify factors significantly related to TNF-α, indicating that there is no over-prediction problem.

## 5 Discussion

Dimensionality reduction is a prevalent preprocessing step in high dimensional microbiome analysis. In this paper, we propose a new GLM-based zero-inflated generalized Poisson factor analysis model to analyze high-dimensional microbiome count data. This method explores the correlation between microbial taxa and response variables, and focuses on selecting a few common factors that summarize the majority of variable information, thus one can mitigate the high dimensionality problem and the computational expenses. The GZIGPFA model can simultaneously consider the zero-inflation, over-dispersion, and high-dimensional characteristics of microbial data. Meanwhile, the model directly models absolute abundance data, avoiding the problem of information loss during data conversion. We establish a link function between generalized Poisson expectation and true zero probability within the GLM framework, and perform parameter estimation using the alternating maximum likelihood algorithm. The rank of the model was determined via cross-validation method. In addition, we performed simulation studies under different scenarios and compared the GZIGPFA method with existing methods to validate the performance of the proposed method. In the analysis of gut microbiome data, the proposed method identified microorganisms significantly associated with obesity.

The novelty of the GZIGPFA method is reflected in the combination of the ZIGP model and the factor analysis model, which provides more possibilities for future microbial-related analysis work. Furthermore, upon review of the existing literature pertaining to microbiome data analysis, our proposed approach represents the inaugural utilization of the zero-inflated generalized Poisson model in microbial datasets, which expands the methodological options of researchers for addressing complex microbiome datasets. In addition to microbiome data, the proposed method can be used for other count data such as micro RNA data, single-cell RNA-seq data, etc. In addition, other suitable models can be extended to the framework of this article to provide more statistical methods for the analysis of high-dimensional microbiome data in the future.

The work presented in this paper remains subject to certain limitations. In this paper, a cross-validation method is used for rank estimation, which is accompanied by a high computational cost, although the results have high accuracy. In future work, the process of rank estimation can be further optimized to improve computational efficiency. In addition, the GZIGPFA model proposed in this article can only extract common factors associated with obesity phenotypes from numerous microbial taxa. The meaning of common factors needs to be determined based on existing prior information, and the interpretation of the actual meaning of each factor is not absolute. We can further extend our approach to provide a more comprehensive tool for the analysis of microorganisms in the future. Finally, although the performance of the log-PCA method in real data analysis closely resembles that of our method, it employs a strategy of replacing zeros in the data with pseudo counts. However, there is no consensus on how to choose the pseudo count, and it has been shown that the choice of pseudo count can affect the conclusions of a microbiome analysis (Costea et al., 2014; Paulson et al., 2014). The gut microbiome data that we used in real data analysis contains ~45% zeros, which is moderately zero-inflated. Perhaps the strategy of replacing zeros has less impact on the results, which may be the main reason why we did not show a clear advantage. Once a dataset shows a serious zero-inflated trend, the log-PCA method may become unstable. In the field of microbiology, it is common for microbiome data to be severely zero-inflated (Paulson et al., 2013; Silverman et al., 2020). Due to sharing restrictions on these data, we do not conduct a practical demonstration in this article.

## Data availability statement

The mouse gut microbiome dataset for the real data analysis section was obtained with the support of Professor Qingshen Sun. The datasets presented in this article are not readily available because they they have not been made publicly available by Sun et al. (2019). Requests to access these datasets should be directed to corresponding author JC, jinlingchi_edu@163.com.

## Ethics statement

The manuscript presents research on animals that do not require ethical approval for their study.

## Author contributions

JC: Software, Visualization, Writing—original draft, Writing—review & editing, Methodology. JY: Funding acquisition, Writing—review & editing. YZ: Data curation, Funding acquisition, Writing—review & editing.
