# Supplementary material for: A GLM-based zero-inflated generalized Poisson factor model for analyzing microbiome data
Source: Front Microbiol. 2024 May 30;15:1394204. doi: 10.3389/fmicb.2024.1394204 (PMC11173601; doi:10.3389/fmicb.2024.1394204)
Supplement: Supplementary file 1 [file Data_Sheet_1.pdf]

Supplementary Material

A APPENDIX A

The details of the EM algorithm

For the convenience of subsequent algorithm description, we can denote $\boldsymbol{y}_j$ or $\boldsymbol{y}_{(i)}$ in step 1 or step 2 simply as $\boldsymbol{y}$. Let the response variable be $\boldsymbol{y} = (y_1, \ldots, y_i, \ldots, y_n)^T$ follow a ZIGP distribution:

$$y_i \sim \begin{cases} 0, & \text{with probability } \phi_i, \\ GP(T_i\lambda_i, \alpha), & \text{with probability } 1-\phi_i, \end{cases}$$

where T_i is the relative library size; λ_i is an element in $\boldsymbol{\lambda}_j$ (step 1) or $\boldsymbol{\lambda}_{(i)}$ (step 2); the ϕ_i is an element in $\boldsymbol{\phi}_j$ (step 1) or $\boldsymbol{\phi}_{(i)}$ (step 2).

For simplicity, we harmonize the notation of the regression models in steps 1 and 2 in the description of the EM algorithm. Let $\boldsymbol{X}$ be a covariate matrix and $\boldsymbol{\beta}$ be the regression coefficient vector to be estimated. Then,

$$\log(\lambda_i) = \boldsymbol{x}_i^T\boldsymbol{\beta},$$
$$\text{logit}(\phi_i) = -\tau\log(\lambda_i),$$

where column vector $\boldsymbol{x}_i$ denotes the i-th row of matrix $\boldsymbol{X}$. In step 1, we assume that the covariate matrix takes the factor score matrix, i.e., $\boldsymbol{X} = \boldsymbol{F}^{old}$, and the regression coefficient vector $\boldsymbol{\beta}$ corresponds to the factor loading vector $\boldsymbol{l}_{(j)} = (l_{j1}, l_{j2}, \ldots, l_{jK})$. In step 2, we assume that the covariate matrix takes the factor loading matrix, i.e., $\boldsymbol{X} = \boldsymbol{L}^{new}$, and the regression coefficient vector $\boldsymbol{\beta}$ corresponds to the factor score vector $\boldsymbol{f}_{(i)} = (f_{i1}, f_{i2}, \ldots, f_{iK})$.

To estimate the parameter vector $\boldsymbol{\theta} = (\tau, \alpha, \boldsymbol{\beta})^T$, we should maximize the likelihood function. However, the explicit solution of each parameter cannot be obtained by directly using the maximum likelihood estimation method. Therefore, we perform parameter estimation of the regression model with the EM algorithm.

The zeros in the microbiome data may be attributed to either the absence of taxa in the sample or technical reasons that result in some taxa not being detected. Thus, we introduced the latent variables $\boldsymbol{z} = (z_1, z_2, \ldots, z_n)^T$, where z_i indicates whether y_i is from excess zeros:

$$z_i = \begin{cases} 1, & y_i \text{ from excess zeros}, \\ 0, & y_i \text{ from GP distribution}. \end{cases}$$

The complete likelihood function is as follows:

$$L(\boldsymbol{\theta}|\boldsymbol{y}, \boldsymbol{z}, \boldsymbol{X}) = \prod_{i=1}^{n} \phi_i^{z_i} \left\{ (1-\phi_i) \left[\frac{1}{y_i!} \left(\frac{T_i\lambda_i}{1+\alpha T_i\lambda_i} \right)^{y_i} (1+\alpha y_i)^{y_i-1} \exp\left(-\frac{T_i\lambda_i(1+\alpha y_i)}{1+\alpha T_i\lambda_i} \right) \right] \right\}^{1-z_i}. \tag{A.1}$$

With the EM algorithm, the estimation of unknown parameters can be conducted in two steps. First, the E-step is to replace latent variable z_i with its conditional expectations

$$
\begin{aligned}
w_i &= E(z_i|\tau, \alpha, \boldsymbol{\beta}, \boldsymbol{y}, \boldsymbol{X}) \\
&= \begin{cases} \frac{1}{1+\exp(\tau \boldsymbol{x}_i^T \boldsymbol{\beta} - \frac{T_i\lambda_i}{1+\alpha T_i \lambda_i})}, & \text{if } y_i = 0, \\ 0, & \text{if } y_i \neq 0, \end{cases}
\end{aligned}
$$

Then, replacing z_i in formula (A.1) with its conditional expectation, the complete log-likelihood function can be written as:

$$
\begin{aligned}
\ln L(\boldsymbol{\theta}|\boldsymbol{y}, \boldsymbol{w}, \boldsymbol{X}) =& \sum_{i=1}^{n} w_i \log(\phi_i) + \sum_{i=1}^{n} (1-w_i) \left[\log(1-\phi_i) + y_i \log\left(\frac{T_i\lambda_i}{1+\alpha T_i \lambda_i} \right) \right. \\
& \left. + (y_i - 1)\log(1+\alpha y_i) - \log(y_i!) - \frac{T_i\lambda_i(1+\alpha y_i)}{1+\alpha T_i\lambda_i} \right].
\end{aligned} \tag{A.2}
$$

Next, the M-step is to find the optimal solution that maximizes the conditional expectation of the complete log-likelihood function $\ln L(\boldsymbol{\theta}|\boldsymbol{y}, \boldsymbol{w}, \boldsymbol{X})$ with the Levenberg-Marquardt (LM) algorithm. The LM algorithm is utilized to identify optimal solutions because it is robust and efficient in many situations (Moré, 1978). The convergence is evaluated by the Frobenius norm of the difference in $\boldsymbol{\beta}$ between two iterations, with an empirical threshold of 1% (Xu et al., 2020). The detailed procedure of the LM algorithm is given in Appendix B of the Supplementary Material.

The estimates of parameter vector $\boldsymbol{\theta} = (\tau, \alpha, \boldsymbol{\beta})^T$ was obtained through the EM algorithm. In step 1, the parameter vector $\boldsymbol{\beta}$ is the corresponding load vector $\boldsymbol{l}_{(j)}$ when the j-th column of matrix $\boldsymbol{Y}$ is used as the response variable. Then, the factor loading matrix is $\boldsymbol{L}^{new} = (\boldsymbol{l}_{(1)}^T, \boldsymbol{l}_{(2)}^T, \ldots, \boldsymbol{l}_{(m)}^T)^T$. In step 2, the parameter vector $\boldsymbol{\beta}$ is the corresponding factor socre vector $\boldsymbol{f}_{(i)}$ when the i-th row of matrix $\boldsymbol{Y}$ is used as the response variable. Then, the factor score matrix is $\boldsymbol{F}^{new} = (\boldsymbol{f}_{(1)}^T, \boldsymbol{f}_{(2)}^T, \ldots, \boldsymbol{f}_{(n)}^T)^T$.

B APPENDIX B

The details of the Levenberg-Marquardt algorithm

We use the Levenberg-Marquardt algorithm to solve the unknown parameter α, τ and $\boldsymbol{\beta}$. The objective function is $Q(\alpha, \tau, \boldsymbol{\beta}) = -\ln L(\boldsymbol{\theta})$, and we want to minimize the objective function. The log likelihood function is:

$$
\begin{aligned}
\ln L(\boldsymbol{\theta}) =& \sum_{i=1}^{n} w_i \log(\phi_i) + \sum_{i=1}^{n} (1-w_i)\log(1-\phi_i) \\
& + \sum_{i=1}^{n} (1-w_i) \Big[y_i \log(T_i\lambda_i) - y_i \log(1+\alpha T_i\lambda_i) \\
& + (y_i - 1)\log(1+\alpha y_i) - \frac{T_i\lambda_i(1+\alpha y_i)}{1+\alpha T_i\lambda_i} - \log(y_i!) \Big].
\end{aligned}
$$

The first and second derivative of $\ln(L)$ are:

$$J_\tau = \frac{\partial \ln(L)}{\partial \tau} = \sum_{i=1}^{n}(r_i - w_i)\boldsymbol{x}_i^T\boldsymbol{\beta},$$

$$J_\alpha = \frac{\partial \ln(L)}{\partial \alpha} = \sum_{i=1}^{n}(1 - w_i)\left[-2s_iy_i + s_i^2(1+\alpha y_i) + \frac{y_i(y_i-1)}{1+\alpha y_i}\right],$$

$$J_\beta = \frac{\partial \ln(L)}{\partial \boldsymbol{\beta}^T} = \sum_{i=1}^{n}\left\{\tau(r_i - w_i) + (1-w_i)\Big[y_i - (1+2\alpha y_i)s_i + \alpha(1+\alpha y_i)s_i^2\Big]\right\}\boldsymbol{x}_i^T.$$

$$H_{\tau\alpha} = \frac{\partial^2 \ln(L)}{\partial \tau \partial \alpha} = 0,$$

$$H_{\tau\tau} = \frac{\partial^2 \ln(L)}{\partial \tau \partial \tau} = \sum_{i=1}^{n}(r_i^2 - r_i)(\boldsymbol{x}_i^T\boldsymbol{\beta})^2,$$

$$H_{\tau\beta} = \frac{\partial^2 \ln(L)}{\partial \tau \partial \boldsymbol{\beta}^T} = \sum_{i=1}^{n}\Big[r_i - w_i - \tau\boldsymbol{x}_i^T\boldsymbol{\beta}(r_i - r_i^2)\Big]\boldsymbol{x}_i^T,$$

$$H_{\alpha\alpha} = \frac{\partial^2 \ln(L)}{\partial \alpha \partial \alpha} = \sum_{i=1}^{n}(1-w_i)\left[3s_i^2y_i - 2s_i^3(1+\alpha y_i) - \frac{y_i^2(y_i-1)}{(1+\alpha y_i)^2}\right],$$

$$H_{\alpha\beta} = \frac{\partial^2 \ln(L)}{\partial \alpha \partial \boldsymbol{\beta}^T} = \sum_{i=1}^{n}2(1-w_i)\Big[-s_iy_i + (1+2\alpha y_i)s_i^2 - \alpha(1+\alpha y_i)s_i^3\Big]\boldsymbol{x}_i^T,$$

$$\begin{aligned} H_{\beta\beta} = \frac{\partial^2 \ln(L)}{\partial \boldsymbol{\beta} \partial \boldsymbol{\beta}^T} =& \sum_{i=1}^{n}\Big\{\tau^2(r_i^2 - r_i) + (1-w_i)\big[(1+2\alpha y_i)(\alpha s_i^2 - s_i) \\ &+ 2\alpha(1+\alpha y_i)(s_i^2 - \alpha s_i^3)\big]\Big\}\boldsymbol{x}_i\boldsymbol{x}_i^T. \end{aligned}$$

where

$$r_i = \frac{1}{1+\exp(\tau\boldsymbol{x}_i^T\boldsymbol{\beta})},$$

$$s_i = \frac{T_i\exp(\boldsymbol{x}_i^T\boldsymbol{\beta})}{1+\alpha T_i\exp(\boldsymbol{x}_i^T\boldsymbol{\beta})}.$$

Correspondingly, the Jacobian ($\boldsymbol{J}$) and Hessian ($\boldsymbol{H}$) matrix of objective function $Q(\alpha, \tau, \boldsymbol{\beta})$ are:

$$J(\tau, \alpha, \boldsymbol{\beta}) = -\begin{pmatrix} J_\tau \\ J_\alpha \\ J_\beta \end{pmatrix}, \quad H(\tau, \alpha, \boldsymbol{\beta}) = -\begin{pmatrix} H_{\tau\tau} & H_{\tau\alpha} & H_{\tau\beta} \\ H_{\tau\alpha}^T & H_{\alpha\alpha} & H_{\alpha\beta} \\ H_{\tau\beta}^T & H_{\alpha\beta}^T & H_{\beta\beta} \end{pmatrix}.$$

Then, the iteration formula of LM algorithm is:

$$\boldsymbol{\theta}_{t+1} = \boldsymbol{\theta}_t - [H(\boldsymbol{\theta}_t) + \mu I]^{-1}J(\boldsymbol{\theta}_t),$$

where $\boldsymbol{\theta} = (\alpha, \tau, \boldsymbol{\beta})^T$, t is the number of iterations, μ is the damping parameter, which is utilized to regulate the characteristics of the algorithm:

- When $\mu > 0$, the coefficient $[H(\boldsymbol{\theta}_t) + \mu I]^{-1}$ is guaranteed to be positive definite, thereby ensuring the descent direction of the iteration;
- When μ is enlarged, the LM algorithm degenerates into the gradient descent method: $\boldsymbol{\theta}_{t+1} = \boldsymbol{\theta}_t - \frac{1}{\mu} J(\boldsymbol{\theta}_t)$;
- When μ is reduced, the LM algorithm degenerates into the Gauss-Newton method: $\boldsymbol{\theta}_{t+1} = \boldsymbol{\theta}_t - H^{-1}(\boldsymbol{\theta}_t) J(\boldsymbol{\theta}_t)$.

In addition, the conditions for the end of the iteration need to be specified (Only one of the conditions needs to be satisfied):

1. The descent gradient is less than a threshold;
2. The difference between $\boldsymbol{\theta}_{t+1}$ and $\boldsymbol{\theta}_t$ is less than a certain threshold;
3. Reach the maximum number of iterations $t_{\max}$.

Finally, the specific process of the LM algorithm is given in the "Algorithm 3" box.

Algorithm 3 Levenberg-Marquardt algorithm

begin
  $t := 0 \quad \nu := 2 \quad \boldsymbol{\theta} := \boldsymbol{\theta}_0$
  $\boldsymbol{H} := H(\boldsymbol{\theta}) \quad \boldsymbol{J} := J(\boldsymbol{\theta})$
  $condition := (\|J\|_\infty < \varepsilon) \quad \mu := \rho \cdot \max\{H_{ii}\}$
  while (**not** $condition$) **and** ($t < t_{\max}$) **do**
    $t := t + 1$ Solve $(\boldsymbol{H} + \mu I)h = -\boldsymbol{J}$
    $\boldsymbol{\theta}_{t+1} = \boldsymbol{\theta}_t + h$
    $\delta = \frac{Q(\boldsymbol{\theta}_t) - Q(\boldsymbol{\theta}_{t+1})}{h^T J + \frac{1}{2} h^T (H + \mu I) h}$
    if $\delta > 0.001$ **then**
      $\boldsymbol{\theta} := \boldsymbol{\theta}_{t+1} \quad \boldsymbol{H} := H(\boldsymbol{\theta}) \quad \boldsymbol{J} := J(\boldsymbol{\theta}) \quad condition := (\|\boldsymbol{J}\|_\infty < \varepsilon)$
      $\mu := \mu \cdot \max\left\{\frac{1}{3}, 1 - (2\delta - 1)^3\right\}, \quad \nu := 2$
    else
      $\mu := \mu \cdot \nu, \quad \nu := 2 * \nu$
    end if
  end while
end

REFERENCES

Moré, J. J. (1978). The levenberg-marquardt algorithm: Implementation and theory. *Lecture Notes in Mathematics* 630, 105–116

Xu, T., Demmer, R. T., and Li, G. (2020). Zero-inflated poisson factor model with application to microbiome read counts. *Biometrics* 77, 91–101. doi:10.1111/biom.13272
